# A bibliometric analysis of the 100 most-cited articles on curcumin

**DOI:** 10.3389/fphar.2022.963032

**Published:** 2022-08-23

**Authors:** Yan-Xi Zhou, Xiao-Yu Cao, Cheng Peng

**Affiliations:** ^1^ State Key Laboratory of Characteristic Chinese Medicine Resources in Southwest China, College of Pharmacy, Chengdu University of Traditional Chinese Medicine, Chengdu, China; ^2^ Library, Chengdu University of Traditional Chinese Medicine, Chengdu, China

**Keywords:** curcumin, most-cited articles, bibliometric analysis, Web of Science, VOSviewer

## Abstract

**Background**: Extensive studies related to curcumin were carried out over the preceding several decades. Citation frequencies represent the most prominent contributions in a specific field. This research aimed to identify and analyze the 100 most-cited articles on curcumin and to highlight the most important advances in this field.

**Methods:** Highly cited articles were identified in the Web of Science core collection database. “curcumin*” was used as the search string to retrieve in the “Title” field. VOSviewer was applied to perform bibliometric analysis of these papers.

**Results:** Totally 17,645 publications on the topic of curcumin were identified. The top most-cited 100 articles were published between 1973 and 2017. Most of these papers were original (*n* = 62). The total citation frequency in the top 100 article ranged from 355 to 3364, with a median of 560. The United States and India were the major countries researching curcumin. The University of Texas M.D. Anderson Cancer Center was the institution with the highest contribution rate of these articles. The most frequently nominated authors were Aggarwal B. B., Kunnumakkara A. B., Prasad S., and Priyadarsini K. I. The top 100 articles were published in 68 journals. The top four journals in terms of the number of our included articles were *Cancer Research* (*n* = 7), followed by Journal of *Biological Chemistry*, *Biochemical Pharmacology*, and *Cancer Letters*, with 4 articles each. *NF-kappa B*, *cancer*, *gene expression*, *apoptosis*, *inflammation*, *chemopreventive agent*, and *nitric oxide synthase* are presumed to be the current hot topics. Bioavailability, anticancer, anti-inflammatory, and antioxidant activities were the major research directions of curcumin.

**Conclusion:** This study analyzed the 100 most-cited articles on curcumin and provided insights into the characteristics and research hotspots of the articles on this topic.

## Introduction

**FIGURE 1:**
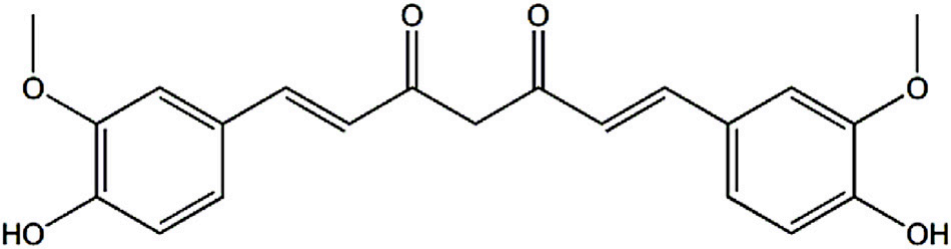
Chemical structure of curcumin.

Curcumin [(1E, 6E)-1,7-bis-(4-hydroxy-3-methoxyphenyl)-1,6-heptadiene-3,5-dione, Figure 1] is a symmetric molecule, also known as diferuloylmethane, with molecular formula C_21_H_20_O_6_, and molecular weight of 368.38 (Priyadarsini, 2014). It is isolated of turmeric (Jianghuang in Chinese, haldi in Hindi, and ukon in Japanese) (Sharma, et al., 2005; Jiang, et al., 2017). Turmeric is derived from the rhizome of the plant *Curcuma longa* L., a perennial herb of the ginger family (Zingiberaceae) that grows extensively in tropical climate (Sharma, et al., 2005; Kocaadam and Şanlier, 2017). India is the initial exporter, and it is also cultivated in Bangladesh, China, Indonesia, Jamaica, and Peru (Zanwar, et al., 2013). Turmeric has been used for a variety of purposes for thousands of years. As a popular oriental spice, it is used to flavor and color food preparations, and is one of the major ingredients of curry powder. Furthermore, it is frequently used in Asian cuisine, especially Indian, Thai, and Pakistani cuisine (Gupta, et al., 2013; Prasad, et al., 2014). In Japan, it is popular as a tea in certain areas, especially in Okinawa (Gupta, et al., 2013). Besides, it is used in cosmetics and as a dye for fabrics such as wool, silk, and cotton (Remadevi, et al., 2007). In addition to the above applications, turmeric has been widely used in Asian countries to treat various diseases for at least 2,500 years (Kocaadam and Şanlier, 2017). Moreover, its use is becoming more and more popular worldwide. For example, in western countries, it is used in mustard blends, sauces, and pickles (Gupta, et al., 2013). In the United States, turmeric is a top selling dietary supplement and the demand for turmeric is steadily increasing (Skiba, et al., 2018). Most of the turmeric consumed in the United States is imported from Asia. However, due to concerns about the quality and production methods of raw materials, the United States began to grow it domestically. And it has been successfully grown in Alabama since 2006 (Shannon, et al., 2019; Setzer, et al., 2021).

The most active ingredient of turmeric is curcumin, which typically accounts for 2%–5% of its content (Gupta, et al., 2013). However, a recent study demonstrated that the curcumin content of Uttarakhand Himalayan turmeric ranged from 6.7% to 10.6% at altitudes of 1,400–1700 m (Thapliyal, et al., 2022). Curcumin was first discovered two centuries ago, and its pure compound was obtained in 1842 (Prasad, et al., 2014). The exact chemical structure of curcumin was revealed in 1910. Then, the synthesis of curcumin was reported in 1913, and chromatography was used to isolate and quantify components of curcumin in 1953 (Prasad, et al., 2014; Li, et al., 2020). Curcumin is a crystalline compound with a brilliant orange-yellow color, which is responsible for the intense yellow color of turmeric (Tønnesen and Karlsen, 1985; Li, et al., 2020). Moreover, curcumin is a lipophilic polyphenol, almost insoluble in water, but soluble in methanol, ethanol, acetone, and dimethylsulfoxide (Aggarwal, et al., 2003; Goel, et al., 2008). Besides, curcumin has significant pharmacological activities, including anti-inflammatory (Jurenka, 2009), antioxidant (Abrahams, et al., 2019), anticancer (Vallianou, et al., 2015), and anti-microbial effects (Sarkar, et al., 2016). Experimental and clinical investigations have reported that curcumin is used in the prophylaxis and treatment of various diseases, such as Alzheimer’s disease (Goozee, et al., 2016), cardiac diseases (Jiang, et al., 2017), diabetes (Nabavi, et al., 2015), rheumatoid arthritis (Mohammadian Haftcheshmeh, et al., 2021), cancer (Mbese, et al., 2019), and psoriasis (Bilia, et al., 2018). Furthermore, clinical studies in humans have indicated that curcumin is generally safe, even at high doses of up to 12 g per day for 3 months (Goel, et al., 2008). Nevertheless, poor aqueous solubility and low bioavailability of curcumin are a matter of concern for researchers. The reasons for its low bioavailability include low solubility (<8 μg/ml in water), poor permeability and absorption, and fast metabolism (short elimination half-life < 2 h) (Nangia and Kuthuru, 2018). To overcome the above factors, a great deal of studies have focused on the synthesis of curcumin and its derivatives as well as the development of drug delivery systems to improve bioavailability and pharmacological activities (Wanninger, et al., 2015; Li, et al., 2021). Among them, nanotechnology, liposome encapsulation and various other preparation techniques are utilized (Li, et al., 2005; Mohanty, et al., 2012; Nangia and Kuthuru, 2018). Recently, an interesting study demonstrated the development of a novel catalyst for ethanol fuel cells by combining curcumin and gold nanoparticles (Sai, et al., 2022).

**FIGURE 2:**
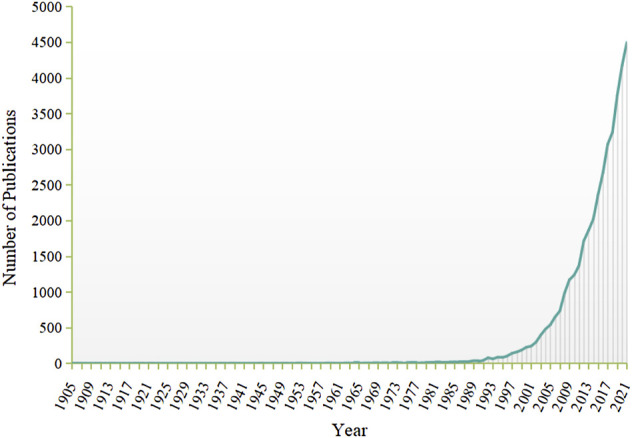
Trends of curcumin in scientific research. Documents deposited in the WOS database from 1900 to 2021, using “curcumin*” as search term in the “Topic” field.

Overall, curcumin has been the research subject of a large number of paper over the past decades. Figure 2 shows the trend of curcumin in scientific research. The earliest record of publications on curcumin in the WOS database is 1905. On the whole, there is an upward trend in the number of papers, indicating an increasing interest in curcumin research. However, the rapid growth of articles makes it difficult to identify the developments and truly influential publications in this area. Bibliometric analysis is a powerful tool for basic science and clinical researchers to access a broad range of knowledge or try to focus on a specific theme, especially when faced with a growing number of publications (García-Fernández, et al., 2020). Citation frequency represents the most important contribution in certain field (Arshad, et al., 2020). Citation analysis is a method of bibliometric analysis in which articles or journals are evaluated and ranked according to the basis citation count (Latif, et al., 2018). Identification milestones in a certain field can be accomplished by analyzing the highly cited studies, especially by analyzing the 100 top-cited studies (Zhang, et al., 2019). The main objective of the current study is to identify the 100 most-cited articles on curcumin and highlight the most notable advances made in the field over the past few decades.

## Materials and methods

### Search strategy

Relevant literatures were retrieved from the Web of Science (WOS) core collection database on 20 April 2022. The search strategy was “Title = curcumin*”. The time covered in WOS core collection database is between 1900 and 2022. No limitations were imposed on publication language, publication year, or publication type. The retrieved literatures were listed in descending order according to the number of citations.

### Literature inclusion

Double-evaluation method was used, with which two independent researchers evaluated each identified literature to ensure the title contained the word “curcumin” or its derivatives and its content was relevant to curcumin. Meanwhile, retracted articles were excluded. In the end, 100 related literatures with the most citations were obtained.

### Data extraction and analysis

VOSviewer 1.6.17 and Microsoft Excel 2013 software were used to analyze and visualize the articles on curcumin. The following information was extracted and analyzed for the included papers: article title, language, publication type, publication year, citation frequency, country, institutions, author, journal, and keywords.

## Results

### Language, publication type, and publication year analysis

**FIGURE 3:**
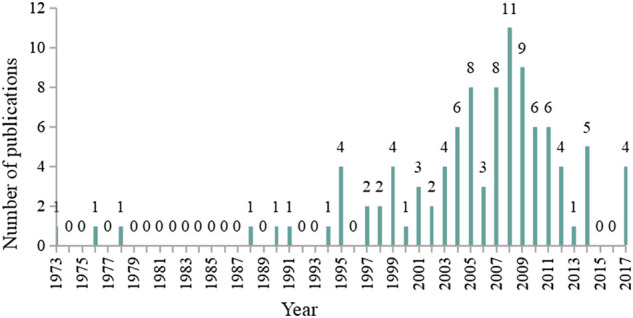
Annual number of publications of the 100 most-cited articles on curcumin.

From the WOS Core Collection Database, a total of 17,645 articles were retrieved using the search term “curcumin*” in the “Title” field. The top 100 articles with the highest cited frequency were all published in English. As for the publication type, the articles could be divided into the following categories: original article (*n* = 62), review (*n* = 34), note (*n* = 2), editorial material (*n* = 1), and letter (*n* = 1). The distribution of the number of publications by year is shown in Figure 3. The top 100 articles with the highest cited frequency were published between 1973 and 2017. From 1973 to 1994, the number of the 100 most cited articles was 0 or 1, and began to be more than 1 since 1995. Furthermore, most articles were published in 2008 (*n* = 11), followed by 2009 (*n* = 9), 2005 (*n* = 8), and 2007 (*n* = 8).

### Citation analysis

**FIGURE 4:**
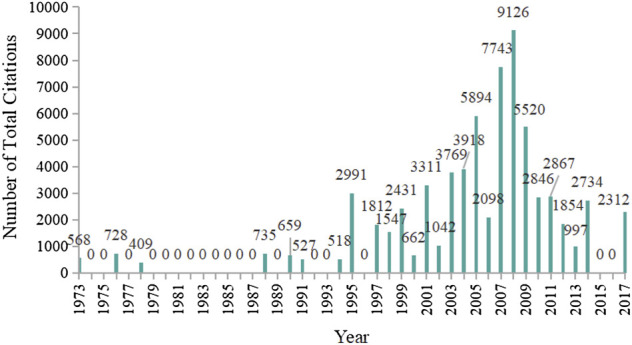
Per year total citations of the 100 most-cited articles on curcumin.

In the 100 most-cited articles, the total citation frequency of each article ranged from 355 to 3364, and the median number of citations was 560. The average citation frequency was 696.18, thirty-one articles were cited more than 700 times, and fifteen articles were cited more than 1,000 times. The article with the highest citation frequency, “Bioavailability of curcumin: Problems and promises,” published in *Molecular Pharmaceutics* in 2007 was cited 3,364 times. The trend in the total number of citations by year is shown in Figure 4. The year with the highest total number of citations was 2008 with 9,126 citations, followed by 2007 with 7,743 citations, 2005 with 5,894 citations, and 2009 with 5,520 citations.

### Country and institution analysis

**FIGURE 5:**
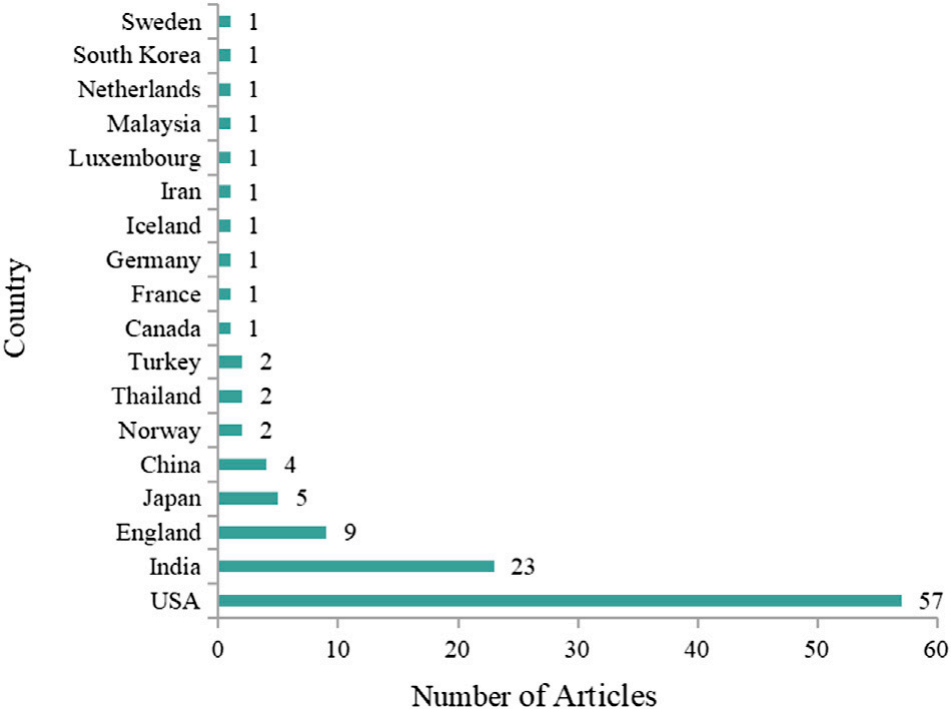
Geographical distribution of the 100 most-cited articles on curcumin.

In terms of the geographical distribution, the 100 most-cited articles were attributed to authors from 18 countries, as shown in Figure 5. The United States was the most productive country with 57 articles, followed by India with 23 articles and England with 9 articles. According to VOSviewer analysis, a total of 120 institutions contributed to the 100 most-cited articles on curcumin, and the institutions with publication quantity ≥ 3 are listed in Table 1. Of the seven institutions, three were from the United States, two from India, and 1 each from England and China. The University of Texas M.D. Anderson Cancer Center was the largest contributor in terms of number of publications (*n* = 27), followed by the University of Leicester (*n* = 6), Bhabha Atomic Research Center (*n* = 5), and University of California, Los Angeles (*n* = 5).

**TABLE 1:** Institutions contributing to the 100 most-cited articles on curcumin (number of articles ≥ 3).

Institution	Country	Number of articles
The University of Texas M. D. Anderson Cancer Center	United States	27
University of Leicester	England	6
Bhabha Atomic Research Center	India	5
University of California, Los Angeles	United States	5
Central Drug Research Institute	India	3
National Taiwan University	China	3
University of South Dakota	United States	3

### Author analysis

The number of scientific papers published by the author, to some extent, represents the author’s contribution and activity in this field (Wu, et al., 2021). A total of 381 authors were involved in the 100 most-cited articles on curcumin, and the authors with the number of published articles ≥ 3 are presented in Table 2. The author who published most articles was Aggarwal BB from the University of Texas M. D. Anderson Cancer Center, whose name appeared in 26 of the top 100 articles. The second most published author, Kunnumakkara AB, came from the same institution, reaching a total of eight articles. The third most published authors were Prasad S and Priyadarsini KI from The University of Texas M. D. Anderson Cancer Center and Bhabha Atomic Research Center, respectively, with five articles each. In addition, there were seven authors who published four articles and 15 authors who published three articles. Of the 26 authors with ≥ 3 documents, 20 were from the United States, three from England, two from China, and one from India.

**TABLE 2:** Authors of the 100 most-cited articles on curcumin (number of articles ≥ 3).

Author	Number of articles	Affiliation	Country
Aggarwal B. B.	26	The University of Texas M. D. Anderson Cancer Center	United States
Kunnumakkara A. B.	8	The University of Texas M. D. Anderson Cancer Center	United States
Prasad S.	5	The University of Texas M. D. Anderson Cancer Center	United States
Priyadarsini K. I.	5	Bhabha Atomic Research Center	India
Anand P.	4	The University of Texas M. D. Anderson Cancer Center	United States
Cole G. M.	4	University of California, Los Angeles	United States
Frautschy S. A.	4	University of California, Los Angeles	United States
Gupta S. C.	4	The University of Texas M. D. Anderson Cancer Center	United States
Shishodia S.	4	The University of Texas M. D. Anderson Cancer Center	United States
Steward W. P.	4	University of Leicester	England
Yang F. S.	4	University of California, Los Angeles	United States
Chauhan S. C.	3	University of South Dakota	United States
Conney A. H.	3	State University of New Jersey	United States
Gescher A. J.	3	University of Leicester	England
Huang M. T.	3	Rutgers State University	United States
Jaggi M.	3	University of South Dakota	United States
Lim G. P.	3	University of California, Los Angeles	United States
Lin J. K.	3	National Taiwan University	China
Newman R. A.	3	The University of Texas M. D. Anderson Cancer Center	United States
Pan M. H.	3	National Taiwan University	China
Patchva S.	3	The University of Texas M. D. Anderson Cancer Center	United States
Sharma R. A.	3	University of Leicester	England
Sundaram C.	3	The University of Texas M. D. Anderson Cancer Center	United States
Sung B.	3	The University of Texas M. D. Anderson Cancer Center	United States
Takada Y.	3	The University of Texas M. D. Anderson Cancer Center	United States
Yallapu M. M.	3	Sanford Research/University of South Dakota	United States

### Journal analysis

In total, the 100 most-cited articles on curcumin were published in 68 journals (Table 3). The journal with the highest number of publications was *Cancer Research* (*n* = 7), followed by Journal of *Biological Chemistry*, *Biochemical Pharmacology*, and *Cancer Letters*, with four articles each. The impact factors (IFs) of these journals ranged from 1.102 to 47.728 (median 5.279); among them, *Science* had the highest value, followed by *Molecular Cancer* (27.401), and *Blood* (23.629). Furthermore, 14 of the top 100 studies were published in journals with an IF more than 10, and 40 were published in journals with an IF more than 5.

**TABLE 3:** Journals of the 100 most-cited articles on curcumin.

Journal	IF^a^	Number of articles
Cancer research	12.701	7
Journal of biological chemistry	5.157	4
Biochemical pharmacology	5.858	4
Cancer letters	8.679	4
Biomaterials	12.479	3
Clinical cancer research	12.531	3
International journal of pharmaceutics	5.875	3
Anticancer research	2.48	2
Advances in experimental medicine and biology	2.622	2
Journal of neuroscience	6.167	2
Molecular pharmaceutics	4.939	2
Critical reviews in food science and nutrition	11.176	2
Molecular pharmacology	4.436	2
AAPS journal	4.009	2
Free radical biology and medicine	7.376	2
Molecules	4.412	2
Oncogene	9.867	2
Journal of pharmacy and pharmacology	3.765	2
Molecular therapy	11.454	1
Biomed research international	3.411	1
Alternative medicine review	3.833	1
Chemico-biological interactions	5.194	1
Colloids and surfaces B-biointerfaces	5.268	1
Drug metabolism and disposition	3.922	1
Blood	23.629	1
Journal of immunology	5.422	1
Journal of neuroscience research	4.164	1
Archiv der pharmazie	3.751	1
Journal of nutrition	4.798	1
Journal of neurochemistry	5.372	1
Clinical gastroenterology and hepatology	11.382	1
Drug discovery today	7.851	1
Journal of agricultural and food chemistry	5.279	1
Journal of pharmacology and experimental therapeutics	4.03	1
Biotechnology advances	14.227	1
Science	47.728	1
Biochemical and biophysical research communications	3.575	1
Carcinogenesis	4.944	1
British journal of pharmacology	8.74	1
Molecular cancer	27.401	1
Foods	4.35	1
Cellular and molecular life sciences	9.261	1
Annals of the New York academy of sciences	5.691	1
Antioxidants and redox signaling	8.401	1
European journal of cancer	9.162	1
Angewandte chemie-international edition	15.336	1
Clinical and experimental pharmacology and physiology	2.557	1
Nanomedicine: nanotechnology biology and medicine	6.458	1
Journal of colloid and interface science	8.128	1
Biomacromolecules	6.988	1
Planta medica	3.356	1
Cancer	6.86	1
Cancer epidemiology biomarkers and prevention	4.254	1
Life sciences	5.037	1
Natural product reports	13.423	1
Food chemistry	7.514	1
European journal of pharmaceutical sciences	4.384	1
Trends in pharmacological sciences	14.819	1
Journal of photochemistry and photobiology C-photochemistry reviews	12.927	1
International journal of biochemistry and cell biology	5.085	1
Cancer research and treatment	4.679	1
Journal of alternative and complementary medicine	2.582	1
Journal of clinical psychopharmacology	3.153	1
Journal of pharmaceutical and biomedical analysis	3.935	1
Acta pharmacologica et toxicologica	—	1
Journal of medicinal chemistry	7.446	1
Current drug targets	3.465	1
Current science	1.102	1

a IFs were from the journal citation report of 2020.

### Article analysis

The details of the top 10 most-cited articles are shown in Table 4. These articles were published between 1997 and 2009, and included 6 reviews, 3 original articles, and 1 editorial material. They were all cited more than 1,200 times. Six of them were published in journals with an IF more than 5. Among the top 10 articles, 3 original articles focused on the mechanisms for treating Alzheimer’s disease (Yang, et al., 2005), phase I clinical trial (Cheng, et al., 2001), and stability and degradation products of curcumin (Wang, et al., 1997), respectively. Four reviews focused on its bioavailability (Anand, et al., 2007), anticancer (Aggarwal, et al., 2003) and other biological activities (Maheshwari, et al., 2006), as well as its potential therapeutic effects on pro-inflammatory diseases (Aggarwal and Harikumar, 2009), respectively. In addition, the other 3 papers reviewed its traditional uses, chemical properties, molecular targets, and clinical studies, etc. (Sharma, et al., 2005; Goel, et al., 2008; Hatcher, et al., 2008).

**TABLE 4:** Top 10 most-cited articles on curcumin.

Title	First author	Year	Number of citations	Journal	IF^a^	Publication type
Bioavailability of curcumin: Problems and promises	Anand P.	2007	3364	Molecular pharmaceutics	4.939	Review
Anticancer potential of curcumin: Preclinical and clinical studies	Aggarwal B.B.	2003	2077	Anticancer research	2.48	Review
Curcumin inhibits formation of amyloid beta oligomers and fibrils, binds plaques, and reduces amyloid *in vivo*	Yang F. S.	2005	1752	Journal of biological chemistry	5.157	Article
Phase I clinical trial of curcumin, a chemopreventive agent, in patients with high-risk or pre-malignant lesions	Cheng A. L.	2001	1653	Anticancer research	2.48	Article
Curcumin as Curecumin: From kitchen to clinic	Goel A.	2008	1523	Biochemical pharmacology	5.858	Editorial material
Curcumin: From ancient medicine to current clinical trials	Hatcher H.	2008	1312	Cellular and molecular life sciences	9.261	Review
Multiple biological activities of curcumin: A short review	Maheshwari R. K.	2006	1269	Life sciences	5.037	Review
Curcumin: The story so far	Sharma R. A.	2005	1225	European journal of cancer	9.162	Review
Potential therapeutic effects of curcumin, the anti-inflammatory agent, against neurodegenerative, cardiovascular, pulmonary, metabolic, autoimmune and neoplastic diseases	Aggarwal B. B.	2009	1216	International journal of biochemistry and cell biology	5.085	Review
Stability of curcumin in buffer solutions and characterization of its degradation products	Wang Y. J.	1997	1208	Journal of pharmaceutical and biomedical analysis	3.935	Article

a IFs were from the journal citation report of 2020.

### Keyword co-occurrence analysis

**FIGURE 6:**
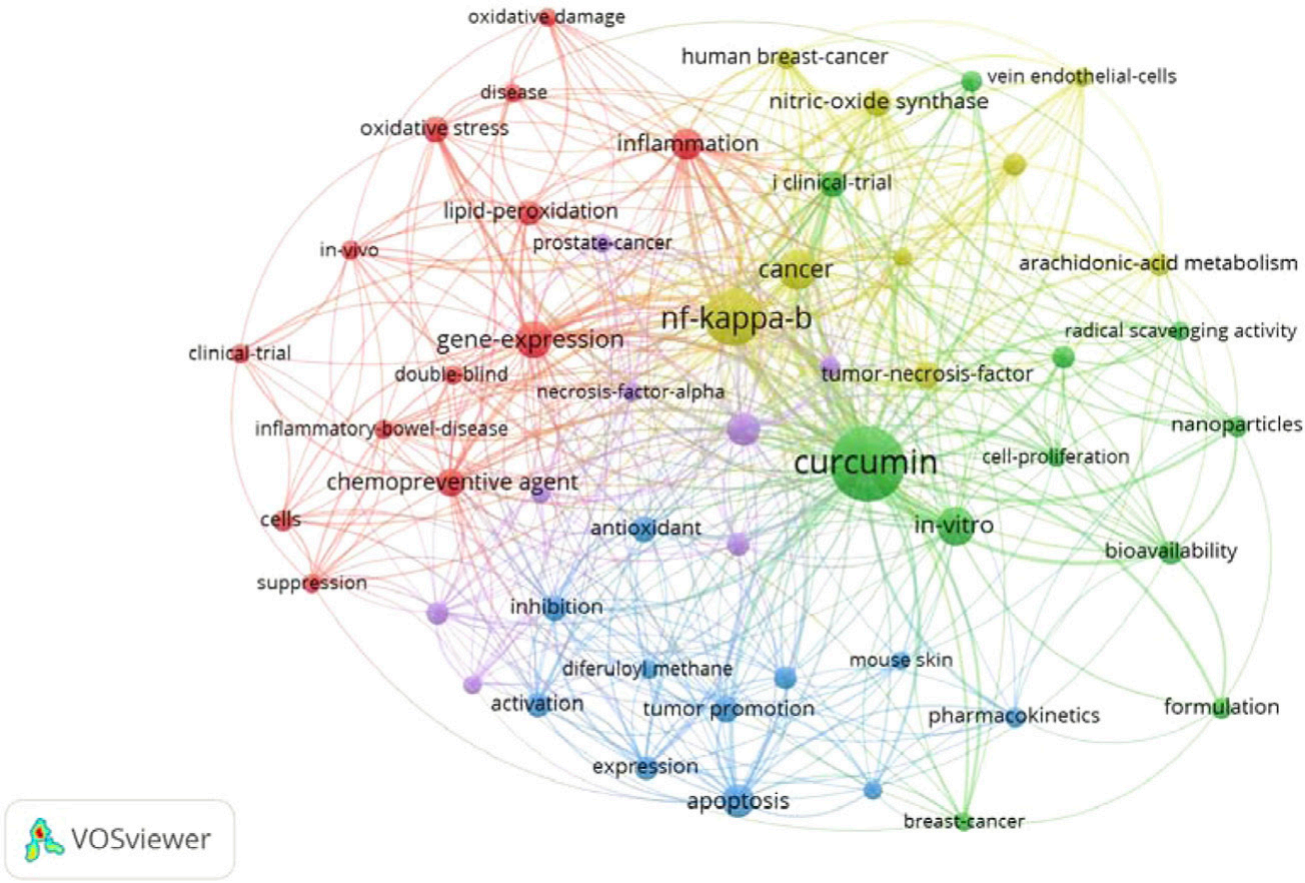
Keyword co-occurrence network visualization of the 100 most-cited articles on curcumin.

VOSviewer software was used to analyze the keywords included in the publications by authors and WOS (KeyWords Plus). A total of 606 keywords were extracted after filtering out keywords with general meaning manually. Only the keywords with a minimum of 4 co-occurrences were visualized, and 52 keywords met the threshold. The network visualization diagram shows the co-occurrence relations of keywords (Figure 6). The size of the circle indicates the number of occurrences of keywords (Wu, et al., 2021). Circles representing keywords such as *curcumin*, *NF-kappa B (NF-κB)*, *cancer*, *in vitro*, *gene expression*, *apoptosis*, *down-regulation*, *inflammation*, *chemopreventive agent*, and *nitric oxide synthase* are larger than others, indicating that these keywords occurred more frequently. Furthermore, all these selected keywords can be roughly divided into into five clusters (red, yellow, green blue, and purple nodes). The top 20 keywords that appeared most frequently are listed in Table 5.

**TABLE 5:** Top 20 keywords with most frequent occurrence.

Keyword	Occurrence	Total link strength
Curcumin	58	242
NF-κB	35	177
Cancer	17	88
*In vitro*	16	53
Gene expression	15	77
Apoptosis	12	48
Down-regulation	12	67
Inflammation	11	56
Chemopreventive agent	9	45
Nitric oxide synthase	9	50
Antioxidant	8	30
I clinical trial	8	44
Inhibition	8	32
Oxidative stress	8	29
Tumor promotion	8	29
Tumor necrosis factor	8	43
Activation	7	23
Bioavailability	7	32
Expression	7	29
Lipid peroxidation	7	28

## Discussion

In this study, the top 100 highly cited articles on curcumin were identified. Using the scientific method of bibliometric and visualized analysis, our study reflected the global trends of the most frequently cited articles in the field of curcumin research. In terms of the publication type, more than 60 percent of the 100 most-cited articles were original articles and one-third were reviews. It is indicated that in the field of research on curcumin, researchers tend to cite original studies rather than review articles or expert opinions. As for publication years, the 100 most cited papers were first published in 1973, 0 or 1 papers were published from 1973 to 1994, more than 1 paper was published since 1995, and the number reached the highest in 2008. Combined with the research trend of curcumin (Figure 2), it is suggested that a large number of studies on curcumin have been carried out since the 1990s, and then abundant high-quality or breakthrough results have been achieved, especially around 2008. According to the number of citations, the most frequently cited articles in the top 100 ones were cited from 355 to 3,364 times. Of the top 100 articles, nearly 60 percent were from the United States, followed by Asian and European countries. Besides, the University of Texas M.D. Anderson Cancer Center, affiliated with the United States, attributed the largest number of publications (*n* = 27). Furthermore, most and the top three productive authors were from the United States. In conclusion, all this information indicated that the United States made a dominant contribution to the development of curcumin research. According to the results of the author analysis, Aggarwal B. B., Kunnumakkara A. B., Prasad S., and Priyadarsini K. I. published the most papers among the top 100 cited articles, indicating that they made significant contributions in this field. This conclusion can be confirmed by searching their names in the “Author” field of multiple databases such as WOS, PubMed, etc. They did publish a large number of articles on curcumin.

A total of 68 journals were involved in this study. Among them, *Science* had the highest IF (47.728), followed by *Molecular Cancer* (27.401), and *Blood* (23.629), with one article published in each of these three journals (Bharti, et al., 2003; Egan, et al., 2004; Wilken, et al., 2011). All journals were ranked in descending order according to the number of the 100 most-cited articles they published. The most published journal was *Cancer Research*, with seven articles, indicating that the research of curcumin in oncology was a hot research direction, and this conclusion could be demonstrated by the fourth published journal (*Cancer Letters*), the second most-cited article (Cheng, et al., 2001), and the third most frequently appearing keyword (*cancer*). In terms of publication type, the top 10 most-cited articles were divided into six reviews, three original articles, and one editorial material. From the content of these articles, it covered a wide range of topics, including the chemical properties, bioavailability, biological activity (especially anticancer activity), mechanism, effect on AD, cancer and pro-inflammatory diseases, and clinical studies of curcumin. Based on the results of keyword co-occurrence analysis and filtering out general meaning keywords, *NF-kappa B (NF-κB)*, *cancer*, *gene expression*, *apoptosis*, *inflammation*, *chemopreventive agent*, and *nitric oxide synthase* were the most commonly used keywords, so these terms are presumed to be the hot spots in this field. Combined with the analysis results of the top 10 most-cited articles and keyword co-occurrence, topics that appeared frequently in curcumin research were as follows: bioavailability, anticancer, anti-inflammatory, and antioxidant activities. By retrieving “Hot Papers”, defined as the top 0.1% of papers in the corresponding academic field in WOS, there were six results on curcumin research, published from May 2020 to February 2022. It can be seen from the contents of several articles that the above-mentioned topics are still hot spots of curcumin research in recent years (Reda, et al., 2020; Abd El-Hack, et al., 2021; Kabir, et al., 2021; Zhang, et al., 2021).

Our initial limitation was conducting the search in the “Title” field. Therefore, literatures that did not contain curcumin in the title were not retrieved or included in our study. Second, we only searched in the WOS core collection database, and not in other databases such as PubMed and Scopus. Finally, given that citations gradually peak within 3–10 years after publication, the current analysis is unable to assess recently published articles (Xie, et al., 2021).

## Conclusion

This bibliometric analysis provides a visualization of the 100 most-cited articles on curcumin. These papers were published between 1973 and 2017, with most published since the year 1995. Meanwhile, the publication types of these articles were mainly original articles. The United States, India, and England contributed the most highly cited papers. The University of Texas M.D. Anderson Cancer Center was the institution with the highest contribution rate of highly cited articles. The most frequently nominated authors were Aggarwal B. B., Kunnumakkara A. B., Prasad S., and Priyadarsini K. I. A total of 68 journals were involved in the 100 top-cited articles. Among them, *Cancer Research* and *Science* were the journals with the largest number of publications and the highest IF, respectively. Keyword co-occurrence analysis indicated that *NF-kappa B (NF-κB)*, *cancer*, *gene expression*, *apoptosis*, *inflammation*, *chemopreventive agent*, and *nitric oxide synthase* were the most frequently used keywords, so these terms are presumed to be the current hot topics. Combined with the results of the article analysis, bioavailability, anticancer, anti-inflammatory, and antioxidant activities were the major research directions of curcumin. This report briefly analyzes the historical development and characteristics of the most frequently cited articles on curcumin, hoping to provide certain perspectives for future research on curcumin or its derivatives.

## Data Availability

The original contributions presented in the study are included in the article/Supplementary Material, further inquiries can be directed to the corresponding author.
